# Supplementary material for: All-at-once RNA folding with 3D motif prediction framed by evolutionary information
Source: Nat Methods. 2025 Oct 3;22(10):2094–106. doi: 10.1038/s41592-025-02833-w (PMC12510878; doi:10.1038/s41592-025-02833-w)
Supplement: Supplementary file 3 — Source code to run CaCoFold-R3D. Data and results. [file 41592_2025_2833_MOESM3_ESM.gz › supplemental_material/results/CaCoFold-R3D/CaCoFold-R3D_Rfam/RF01883_TUG1_2.R2R.sto.pdf]

RF01883_TUG1_2

5′ A A A R U G U G U U G G U C U U U U A G C U C U U U U G U A A U U A A R U C U G G A Y G U A C C U C A A A A G A A Y C U U R A G A C U G U G G U G A U A A G R U G C U U U C C U C
